# Lifting the veil of secrecy: maternal and neonatal outcome of oocyte donation pregnancies in Germany

**DOI:** 10.1007/s00404-021-06264-8

**Published:** 2021-10-04

**Authors:** J. Altmann, J. Kummer, F. Herse, L. Hellmeyer, D. Schlembach, W. Henrich, A. Weichert

**Affiliations:** 1 Department of Obstetrics, Charité—Universitätsmedizin, 10117 Berlin, Germany; 2 Berlin Institute of Health, Berlin, Germany; 3 Department of Obstetrics and Gynecology, Vivantes Hospital Friedrichshain, Berlin, Germany; 4 Experimental and Clinical Research Center—A Joint Cooperation Between the Max-Delbrück-Center for Molecular Medicine and the Charité—Universitätsmedizin, Berlin, Germany; 5 Clinic of Obstetric Medicine, Vivantes Hospital Neukoelln, Berlin, Germany; 6 Center for Prenatal Diagnosis, Bergmannstrasse 102, Berlin, Germany

**Keywords:** Oocyte-donation, Egg-donation, Preeclampsia, Pregnancy risks, Peripartum hemorrhage

## Abstract

**Background:**

In Germany, performing fertility procedures involving oocyte donation is illegal, as stated by the Embryo Protection Law. Nonetheless, in our clinical routine we attend to a steadily rising number of pregnant women, who have sought oocyte donation abroad. Due to the legal circumstances many women opt to keep the origin of their pregnancy a secret. However, studies have shown, that oocyte donation is an independent risk factor for the development of pregnancy complications, such as preeclampsia.

**Objective:**

The aim of this study is to evaluate maternal and neonatal outcomes of oocyte donation pregnancies in three large obstetric care units in Berlin, Germany.

**Methods:**

We retrospectively analyzed all available medical data on oocyte donation pregnancies at Charité University hospital, Vivantes Hospital Friedrichshain, and Neukoelln in the German capital.

**Results:**

We included 115 oocyte donation (OD) pregnancies in the present study. Our data are based on 62 singleton, 44 twin, 7 triplet, and 2 quadruplet oocyte donation pregnancies. According to our data, oocyte donation pregnancies are associated with a high risk of adverse maternal and fetal outcome, i.e., hypertension in pregnancy, preterm delivery, Cesarean section as mode of delivery, and increased peripartum hemorrhage.

**Conclusion:**

Although oocyte donation is prohibited by German law, many couples go abroad to seek reproductive measures using oocyte donation after former treatment options have failed. OD pregnancies are associated with a high risk of preeclampsia, C-section as mode of delivery, and peripartum hemorrhage. Detailed knowledge of the associated risks is of utmost importance to both the patient and the treating physician and midwife.

## Introduction

Since the first successful pregnancy resulting from oocyte donation in 1984 [1] was performed, we are witnessing the success story of oocyte donation (OD) in the field of reproductive medicine worldwide. The procedure of oocyte donation encompasses the hormonal stimulation and retrieval of stimulated follicles in a usually young and healthy woman, who acts as a donor, the insemination with either the partner’s or a donor’s sperm through IVF (in-vitro fertilization) or ICSI (intracytoplasmatic sperm injection) and insertion of the embryo(s) into the recipient’s uterus.

Formerly used to help women with premature ovarian insufficiency, oocyte donation is nowadays used for a number of reasons: inherited diseases, genetic anomalies, or peri- or post-menopausal infertility [2]. Infertility is a multifactorial disorder that affects 2–3% of the population [3]. In a rapidly changing modern society with a steadily increasing life expectancy and long career paths, the issue of age-related infertility is aggravated due to the fact that many women decide to postpone child-bearing. In 2018 the mean age of child-bearing in Germany was 30.0 years; latest data suggest that the number in the German population is still rising [4]. In Germany, around a quarter of women giving birth are over the age of 35 years [4, 5]. The number of children born by women over the age of 40 years is increasing as well, accounting for 43,988 children in 2019 in Germany [4].

According to the European IVF-monitoring Consortium 73,927 treatment cycles using oocyte donation were performed in 2016, leading to 22,497 deliveries in the participating countries [6]. The numbers are continuously rising according to the European Society of Human Reproduction and Embryology [6]. Reproductive procedures involving oocyte donation are illegal in Germany due to the Embryo Protection Law [7]. Therefore, after prolonged unsuccessful cycles of IVF or ICSI in Germany, many couples use oocyte donation abroad as their last resort to fulfill their wish to have children. Cross-border reproductive care is a growing phenomenon, yet little is known about its scope [8].

No data exist on how many German women seek cross-border reproductive care involving oocyte donation driven by legal restrictions in Germany. In our clinical experience in some of the largest obstetric care units in Germany, the number of pregnancies resulting from oocyte donation in Germany rises continuously despite a presumably high number of unrecorded cases. On account of the legal situation in Germany no guidelines or professional recommendations exist on how to monitor these high-risk pregnancies properly. Furthermore, knowledge about the specific risks related to oocyte donation pregnancies is scarce. Additionally, affected women might opt to keep the origin of their pregnancy a secret for fear of legal implications or discrimination. This further increases the risk of not being monitored appropriately.

So far, no studies concerning oocyte donation pregnancies in Germany have been published. Using a retrospective analysis of all available data on oocyte donation pregnancies in three large prenatal and obstetric care units in the German capital, we aim at assessing the course of pregnancy, maternal, and neonatal outcomes within the exceptional circumstances of a country, in which performing oocyte donation is illegal.

## Methods

Prior to the retrospective analysis formal approval by the ethics committee of the Charité University Hospital has been sought (number: EA4/176/19).

A retrospective analysis of available data on oocyte pregnancies was performed at the departments of obstetrics of the Charité University Hospital, Vivantes Hospital Friedrichshain, and Vivantes Hospital Neukoelln, all three of them being located in Berlin.

In 2019, the Charité University Hospital accounted for 5526, the Vivantes Hospital Friedrichshain for 3361, and the Vivantes Hospital Neukoelln for 3202 live births.

Obstetric data and images were retrieved from birth records as well as from Viewpoint obstetric imaging database (GE Healthcare, Solingen, Germany) for further analysis. Medical data at the Charité are stored via the hospital’s software system *SAP*. At the Vivantes Clinics the hospital’s software system *ORBIS* is used to store medical data.

All cases were thoroughly analyzed using all maternal and neonatal medical records available. Data analysis at the Charité University Hospital includes all medical data collected from the 1st of January 2000 to the 31th of August 2020, at Vivantes Hospital Friedrichshain from the 1st of January 2017 to the 31th of December 2019 and from the 1st of January 2013 to the 31th of December 2019 at the Vivantes Hospital Neukoelln.

All data were analyzed and stored in a pseudonymous form. Data analysis was performed using Microsoft Excel, version 2016, and G*Power, version 3.1.9.4 [9].

## Results

**Fig. 1:**
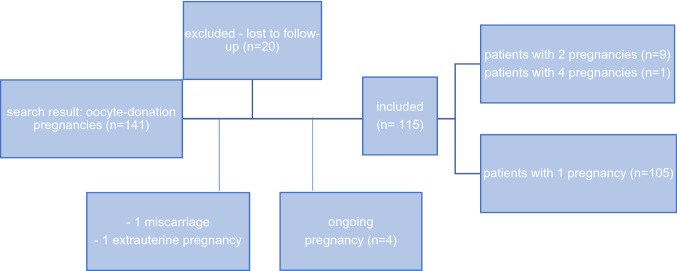
Flow chart of patients included

The initial search revealed 141 pregnancies conceived through oocyte donation (OD). Over the time period of data analysis a steady incline in numbers can be observed. Of those, 4 pregnancies were still ongoing and 20 pregnancies were excluded due to missing follow-up data. Two pregnancies were excluded from the analysis since they resulted in a miscarriage and an ectopic pregnancy. 10 women included in our data had at least two OD pregnancies. These were counted as individual pregnancies (see Fig. 1). Our study collective consists of 62 singleton, 44 twin pregnancies, 7 triplets, and 2 quadruplets. In total, 115 pregnancies and 179 fetuses were included in our retrospective analysis.

### Patient characteristics

The mean maternal age at pregnancy was 44 years, ranging from 29 to 65 years. 68% of women included in the study were within the age cohort of 40 to 50. 16% were below the age of 40 and 13% above the age of 50. The oldest woman included in this study was 65 years old (for patient characteristics see Table 1).

**Table 1:** Patient characteristics (*n*: 115)

Age (years)	Mean 44.17 Range 29–65 SD 7.36
< 40	15.6%
40–44	31.3%
45–49	36.5%
50–54	11.3%
> 55	1.7%
Not known	3.5%
Ethnicity*	
Caucasian	93.9%
Asian	4.3%
African	1.7%
Obstetric history	
Abortion	
1	21.7%
2	11.3%
3	2.6%
> 3	7.8%
Of those > 12. weeks of pregnancy	3.5%
Ectopic pregnancy (at least 1)	7%
Termination of pregnancy	
< 12 weeks of pregnancy (at least 1)	9.5%
> 12 weeks of pregnancy (at least 1)*	2.6%
Genetic disorder of the fetus in previous pregnancy (live birth)	5.2%
Previous termination of pregnancy due togenetic disorder or severe malformations	4.3%
Previous perinatal death of fetus	3.5%
Not known	7%
Parity (only life births)	Mean 1.4 Range 0–13 SD 1.4
None	49.1%
1	39.3%
2	8%
> 3	3.6%
Not known	2.7%
Gynecological disorders	
Fibroids of the uterus	17.6%
Endometriosis	3.7%
Salpingectomy unilateral	1.9%
Salpingectomy bilateral	4.6%
Laparoscopic removal of ovarian cyst	4.6%
Resection of the ovary	0.9%
Prior caesarean section	13.8%
Prior uterine abrasion	13%
Conization	7.4%
Uterus bicornis	1.9%
Isthmocele	0.9%
Not known	6%
Pre-existing conditions	
Mosaic Turner syndrome	0.9%
Hypothyroidism	19.4%
Hyperthyroidism	4.6%
Previous cancer treatment	2.8%
Maternal genetic disorder	3.7%
Diabetes mellitus type 2	2.8%
Hypertension	1.9%
Asthma	1.9%
Depression	1.9%
Ulcerative colitis	1.9%
Coagulation disorder	2.8%
Systemic lupus erythematosus	0.9%
Not known	6%

*SD* standard deviation

*All of those due to genetic disorders or severe malformations

The vast majority of women, 94%, were Caucasian. 4% of women were of Asian and 2% of African descent.

### History of prior pregnancies

Fifty percent of pregnant women had not given birth to a child prior to the current OD pregnancy. 39% of women had given birth once, 8% twice, and 2% at least three times, respectively. The vast majority of these previous pregnancies occurred 10–20 years before the current OD pregnancy.

Within our cohort parity ranged between 0 and 13 former live births. 43% of included women had been pregnant at least once, but miscarried. 12.5% had experienced 3 or more miscarriages. 8% had at least one ectopic pregnancy. In 12% at least one termination of pregnancy had been performed in the past. 4% of women had opted for a termination of pregnancy due to severe malformations or chromosomal aberrations in the past and 5% of women had given birth to a child with genetic disorders, such as trisomy 21 or fragile x syndrome. Another 3.5% had previously lost a child within the perinatal period.

### General medical history

General pre-existing conditions were scarce among the recipients and consisted mainly of hypo- or hyperthyroidism (24%). Other pre-existing disorders included ulcerative colitis, diabetes, asthma, or depression. Inheritable maternal genetic disorders included balanced translocation of chromosomes 1 and 3 as well as fragile x syndrome and were rarely present. One woman suffered from a mosaic form of Turner syndrome.

Several women had either a known gynecological disorder (*n*: 34) or a medical history of surgery on the ovaries, fallopian tubes, uterus, or cervix (*n*: 53). Gynecological disorders consisted mainly of fibroids of the uterus (18%). Endometriosis (4%) was rarely reported. 2% of women had had an unilateral and 5% a bilateral salpingectomy in the past. 13% had a dilatation and curettage in the past, usually in the context of an abortion. 14% of women had delivered by cesarean section prior to the current pregnancy. 7% of women had a conization of the cervix for cervical dysplasia in their medical history. A bicornuate uterus was present in 2%.

Three percent of patients had experienced cancer treatment in the past, including one case of cervical cancer. Among the included patients only one case of premature ovarian insufficiency was observed.

### Data on current pregnancy

Data on fertility procedures were scarce. In the majority of cases oocyte donation and ICSI was performed. At the time of oocyte donation, the donor’s average age was 26 years (range 18–34, SD 3.45). Three women conceived through heterologous insemination in addition to the oocyte donation. In the majority of cases two embryos were transferred. In 23% of pregnancies one embryo was transferred, in 14% three embryos, and in 7% four embryos were transferred (for data on current pregnancy see Table 2).

**Table 2:** Details on current pregnancy (*n*: 115)

Donor age	Mean 26 years Range 18–34 years SD 3.45
Data available	86
Not known	25%
Number of transferred embryos	Mean 2.67 Range 1–4 SD 0.8
1	22.9%
2	55.7%
3	14.3%
4	7.1%
Not known	39.1%
Pregnancies included in the current study	115
Fetuses	179
Singleton	53.9%
Twin	38.3%
Dichorionic	97.7%
Monochorionic diamniotic	2.3%
Triplet	6.1%
Quadruplets	1.7%

*SD* standard deviation

The most common destinations for cross-border reproductive tourism in our cohort are Spain and Czech Republic. Other countries included Cyprus, Russia, Ukraine, Turkey, the United States of America, Iran, Peru, and Ghana. These countries were mostly chosen because of a personal or familial linkage.

The 115 included OD pregnancies comprise 62 singleton pregnancies, 44 twin pregnancies, 7 triplet pregnancies, and 2 quadruplet pregnancy. Two pregnancies were documented former twin pregnancies with the disappearance of one of the fetuses within the first trimester leading to a vanishing twin. The group of 44 twin pregnancies consisted of one was monochorionic diamniotic pregnancy and 43 dichorionic pregnancies.

To enhance comparability, pregnancy outcome data were analyzed within the two major groups: singleton (s) (*n*: 62) and dichorionic twin (t) (*n*: 43) pregnancies. The seven triplet pregnancies and the two quadruplet pregnancies are listed separately (see Table 3).

**Table 3:** pregnancy complications

Data on current pregnancy	Singleton	Twins	Triplets/quadruplets
Adverse outcome			
Selective feticide due to multiple pregnancy	0%	0%	44%
Feticide due to malformations of the fetus	0%	4.5%	0%
Termination of pregnancy due to severe maternal complications	0%	0%	11.1%
IUFD	3.2%	9%	11.1%
Perinatal death due to severe prematurity	3.2%	2.3%	22.2%
Death due to severe malformations during childhood	1.6%	0%	0%
Hypertension in pregnancy			
Pre-existing hypertension	1.6%	2.3%	0%
Pregnancy-induced hypertension	9.7%	2.3%	11.1%
Preeclampsia	16.1%	22.7%	22.2%
HELLP	1.6%	2.3%	0%
Delivery (weeks of gestation)	Mean 36.9 Range 16–41 SD 4.9	Mean 34.9 Range 21–40 SD 4.5	Mean 28 Range 16–37 SD 6.62
Premature delivery	34.5%	63.4%	88.9%
34 + 0 to 37 + 0	13.8%	39%	22.2%
< 34 + 0	20.7%	24.4%	66.7%
Antenatal corticosteroids	16.1%	28.6%	44.4%
Cervical cerclage	8%	4.5%	11.1%
Pessary	1.6%	2.3%	33.3%
Progesterone	1.6%	4.5%	11.1%
Perinatal death due to severe prematurity	1.6%	4.5%	22.2%
Mode of delivery (live births)			
Vaginal delivery	8.1%	9.3%	0%
Vacuum extraction delivery	4.8%	2.3%	0%
Caesarean section	83.9%	88.4%	100%
Planned	35.5%	20.9%	0%
Unplanned or emergency	49%	67.4%	100%
Stillbirth	3.2%	2.3%	33.3%
Peripartum hemorrhage	Mean 683 ml Range 200–3000 ml SD 535	Mean 782 ml Range 300–3000 ml SD 606	Mean 1214 ml Range 400–2500 ml SD 848
> 500 ml blood loss	25%	29%	28.6%
> 1500 ml blood loss	9%	12.9%	28.6%
Total > 500 ml blood loss	34%	41.9%	57.2%
Placental retention > 24 SSW	18.2%	25.8%	28.6%
Placental abruption	0%	3.2%	0%

*IUFD* spontaneous intrauterine fetal death (> 20 weeks of pregnancy), *HELLP* Hemolysis elevated liver enzymes low platelet count, *SD* standard deviation

The only monochorionic diamniotic pair of twins in our cohort led to a twin-to-twin transfusion syndrome at 33 weeks of gestation and subsequent cesarean delivery. After extensive treatment at the neonatal intensive care unit both children were discharged in stable condition.

The group of seven triplet pregnancies consisted of two dichorionic triamniotic and five trichorionic pregnancies. The two quadruplets were tetrachorionic.

### Ultrasound findings

During their pregnancies, all assessed women had detailed I, II, and III trimester screening performed by expert maternal fetal medicine specialists.

The rate of minor fetal anomalies was low: 10 fetuses, 5.6%, of pregnancies exhibited minor anomalies detected in ultrasound or shortly after birth, including inguinal hernia and hydrocele testis.

Pathological findings of the placenta, including bilobed placenta (*n*: 4), placenta previa partialis (*n*: 6), or totalis (*n*: 3) as well as velamentous cord insertion (*n*: 2) were frequently detected. Another sign of the high rate of placental pathology is the high proportion of placental retention (18% in singletons, 26% in twins, 29% in triplets and quadruplets). Retained placenta was defined as the absence of placental expulsion 30 min after delivery of the child.

### Pregnancy-induced hypertensive disorders

Pre-existing hypertension was noted in one singleton and one dichorionic twin pregnancy each. Pregnancy-induced hypertension, a repeatedly measured blood pressure > 140/90 mmHg beginning in the 20th week of pregnancy or above, was observed in six singleton, one twin, and one triplet pregnancy (see Table 3).

Preeclampsia was defined as pregnancy-induced hypertension plus proteinuria > 300 mg in 24 h urine or > 30 mg/mmol protein-to-creatinine ratio or other concomitant neurological or renal signs. The rate of preeclampsia was 16% in singleton, 23% in twin, and 22% in triplet or quadruplet pregnancies. Within the preeclamptic group, mean gestational age at delivery was 35 weeks for singleton and 34 weeks for dichorionic twin pregnancies. The earliest premature delivery due to preeclampsia was at 27 weeks in a dichorionic twin pregnancy.

In one severe case of preeclampsia in a twin pregnancy—after antenatal corticosteroids were applied at 23 weeks of gestation—emergency cesarean section was performed at 26 weeks of gestation after intrauterine fetal death of the leading twin. After a prolonged stay at the neonatal intensive care unit, the second twin was discharged in a reduced, but stable condition with home monitoring and home oxygen supplementation.

Hemolysis, elevated liver enzymes, and low platelet count (HELLP) syndrome was observed in one singleton pregnancy and one twin pregnancy, both were associated with severe preeclampsia.

### Prematurity

In eight patients premature contractions lead to premature cervical ripening and preterm delivery. Premature rupture of the membrane was frequently found as well (*n*: 16). Mean age at delivery was 37, 35, and 28 weeks of gestation for singleton pregnancies, dichorionic twin pregnancies, and the group of triplets and quadruplets (see Table 3). Within our cohort, 21% of singletons, 24% of twins, and 67% of triplets or quadruplets were delivered before the 34th week of gestation was completed. In total, premature delivery < 37 + 0 weeks of gestation accounted for 35% of singleton, 63% of dichorionic twin, and 89% of triplet/quadruplet pregnancies.

Sixteen percent of singletons, 29% of twins, and 44% of triplets or quadruplets received antenatal corticosteroids (12 mg of dexamethasone twice within 24 h) as well as tocolysis for 48 h, usually with nifedipine orally. Surgical cerclage of the cervix, pessary therapy, or medication with progesterone were commonly applied to impede premature delivery (8%, 2%, and 2%, respectively, in singletons; 5%, 2%, and 5%, respectively, in twins; 11%, 33%, and 11%, respectively, in triplets or quadruplets).

### Adverse outcomes

Spontaneous intrauterine fetal death (IUFD) of one fetus occurred in two singleton, four twin, and one triplet pregnancies (singleton: 3.2%, twin: 9%, triplets/quadruplets: 11.1%).

In addition, five perinatal deaths occurred due to severe prematurity (singleton: 3.2%, twin: 2.3%, triplets/quadruplets: 22.2%). In four of these pregnancies age at delivery was between 21 and 23 weeks of gestation causing immediate fetal demise. In case of the singleton pregnancy, the neonate was born at 26 weeks of gestation and died three weeks after birth despite intensive care measures.

In six pregnancies feticides were performed, in two of those due to malformations of the fetus, including microduplication15q11q13 syndrome (twins: 4.5%, triplets/quadruplets: 44%). The remaining four pregnancies were three triplet pregnancies and one quadruplet pregnancy. In three of the trichorionic triplet pregnancies multifetal pregnancy reduction of one fetus was performed. One led to the delivery of two healthy children at 37 weeks of gestation. Another one resulted in stillbirth at 23 weeks of gestation. The other pregnancy was terminated at 17 weeks of gestation—2 weeks after multifetal pregnancy reduction had been performed—due to severe maternal complications through massive loss of weight, hyperemesis, esophagitis, and pregnancy-induced hyperthyroidism. In this case, stillbirth was followed by massive peripartum hemorrhage with 2.5 l of blood loss requiring blood and plasma transfusions.

IUFD occurred in another trichorionic triplet pregnancy, two healthy children were delivered at 35 weeks of gestation.

One dichorionic triamniotic triplet pregnancy resulted in preeclampsia and the delivery of three healthy neonates at 36 weeks of gestation.

The remaining two triplet pregnancies, one trichorionic, and one dichorionic triamniotic pregnancy resulted in premature delivery at 27 and 29 weeks, respectively, with good neonatal outcome.

In one quadruplet pregnancy selective feticide of two fetuses was performed in the 12th week of pregnancy. In this case, selective feticide was requested by the patient on the grounds of the high pregnancy risks associated with multifetal pregnancy. At 23 weeks of gestation premature rupture of the membranes led to chorioamnionitis and subsequent perinatal death of all fetuses.

The other quadruplet pregnancy was affected by severe preeclampsia in 26 weeks of gestation and subsequent C-section. All four neonates survived although all of them were affected by common preterm complications. One neonate was afflicted by necrotizing enterocolitis and consecutive bowel perforation leading to emergency surgery on the 7th day of life. Another neonate suffered from intraventricular hemorrhage on the 3th day of life. He was treated by the surgical implantation of a ventriculoperitoneal shunt system. Today, the 5-year-old boy shows neurological long-term damage, such as hemiparesis and spasticity, due to posthemorrhagic hydrocephalus. The other two boys suffered from sepsis, but were both treated successfully at the neonate intensive care unit.

Overall outcome of the seven triplet pregnancies and the two quadruplet pregnancy was poor; out of 29 only 18 fetuses survived (including feticides) (see Table 3).

### Delivery

The majority of pregnancies were delivered by cesarean section (singleton: 83.9%, twins: 88.4%, triplets/quadruplets: 100%) with a large percentage of unplanned of emergency C-sections (singleton: 49%, twins: 67.4%, triplets/quadruplets: 100%; Table 3). Reasons for planned cesarean section were as follows: Uterine scars due to prior operations, multiple pregnancies, or breech position of the fetus. Unplanned cesarean sections were performed for the following reasons: premature contractions or rupture of the membrane, pathological fetal heart tracing indicating major fetal distress, prolonged or obstructed labor, fetal malposition or malpresentation, IUFD of one fetus in multifetal pregnancies, premature placental abruption, or severe bleeding during childbirth. Four children were delivered using vacuum extraction due to major fetal distress.

3.2% of singleton, 4.5% of twin, and 33.3% of triplet or quadruplet deliveries were stillbirths.

### Peripartum hemorrhage

Retention of the placenta leading to the manual removal of the placenta occurred in 18% of singleton, 26% of twin, and 29% of triplet or quadruplet deliveries (see Table 3).

Increased peripartum blood loss occurred irrespective of multiplicity of the pregnancy. Mean blood loss was 683 ml in singletons (range 200–3000 ml), 782 ml in dichorionic twins (range 300–3000 ml), and 1214 ml in triplets or quadruplets (range 400–2500 ml).

In 39% of pregnancies estimated blood loss was more than 500 ml during delivery. In 9% of singleton, 13% of twin, and 29% of triplet/quadruplet pregnancies peripartum, blood loss was more than 1.5 l necessitating manual techniques, intensified treatment with uterotonic agents, application of tranexamic acid, and/or transfusion of erythrocytes and plasma.

The maximum peripartum blood loss was three liters and occurred in three cases, both in singleton and twin pregnancies. In three cases hemorrhage was so massive that the attending physicians opted for the application of a Bakri balloon and/or tamponade or surgical ligation of the uterine artery. In none of these cases hysterectomy was needed.

### Neonatal outcome

There was a high rate of adverse pregnancy outcomes, especially affecting triplets and the quadruplet pregnancy (see Table 4 for neonatal outcome).

Mean weight at birth was 2803 g in singleton and 2266 g in twin pregnancies. In 11% of singleton and 19% of twin pregnancies birth weight was below the 10th percentile and 8% or 2%, respectively, were above the 90th percentile.

APGAR values below 7 at 5 and 10 min after birth occurred rarely and most neonates adapted quickly (singleton: 6.4%, twins: 10.6%).

The vast majority of neonates presented with a pH of the umbilical cord artery within the normal ranges above 7.2 (93% of singletons, 89% of twins).

## Discussion

In our cohort, patients using cross-border fertility treatment involving oocyte donation were on average 44 years old and had a history of at least one miscarriage, ectopic pregnancy, or termination of pregnancy. 13% of assessed women were above the age of 50. The oldest woman included in the current study was 65 years old. A Spanish study found significant differences between German, French, and Italian couples seeking cross-border reproductive care involving oocyte donation [10]. In comparison to French and Italian women, most German women had already undergone more than three unsuccessful IVF or ICSI cycles [10, 11] and were older than couples from France or Italy. Comparing our data to the literature, mean age at pregnancy within our cohort of patients seems to be higher than in Spanish, Italian, or French women undergoing oocyte donation (38–41 years) [10]. Although studies have revealed that oocyte donation is an independent risk factor for pregnancy complications [12], age-related implications might contribute to the high number of complications within our study population [13].

Half of the women had given birth to at least one child, usually 10–20 years before, and chose to have another child over the age of 40. Judging from the available data, genetic disorders, premature ovarian insufficiency, or previous cancer treatment are rare. Instead, age-related infertility was the main reason for the use of oocyte donation.

As a result of age-related risks, but also moral implications, several countries have opted to implement an upper age limit to financial reimbursement of fertility procedures, for example, until the age of 43 in France [2, 13].

In 56% of pregnancies two embryos from an—on average—26 years old donor were transferred into the recipient’s uterus. As a result, multiple pregnancies after oocyte donation are rather the rule than the exception. In 14% three embryos and in 7% of patients four embryos were transferred resulting in seven triplets and one quadruplet pregnancies. The vast majority used the partner’s sperm for fertilization. Heterologous insemination has been rarely used. It is noteworthy that studies have shown that sperm from an unfamiliar donor increases the risk of preeclampsia further [14, 15].

Triplet and quadruplet pregnancies are burdened with a particularly high risk of adverse maternal and/or fetal outcome. After returning from abroad half of these women opted for multifetal pregnancy reduction of one or two fetuses to reduce the risks associated with triplet or quadruplet pregnancies. Thus, detailed counseling about potential risks of multifetal pregnancies is highly recommended prior to the insertion of several donated oocytes. As shown above, selective feticide is associated with a high risk of fetal death of another fetus or late miscarriage of the pregnancy. Of 29 fetuses included in the group of triplet and quadruplet pregnancies only 18 fetuses survived. In the literature, outcome data on triplet or even quadruplet OD pregnancies are scarce. Only few case series have been published, indicating that obstetric complications, including preeclampsia, gestational diabetes, and preterm delivery, are high [16]. Selective feticide is commonly performed in triplets or quadruplet pregnancies [16].

Since the turn of the century, a change of strategies has taken place in reproductive medicine away from a transfer of many embryos—guaranteeing a high rate of pregnancies with consecutive collateral damage—toward a transfer of only one or two well-developed blastocysts—a lower rate of, but healthy, pregnancies [6]. In a few countries, however, including Bosnia-Herzegovina, Lithuania, and Serbia, more than 40% of transfers are still performed using three embryos [6]. As a consequence of the per se increased risks of OD pregnancies a single-embryo transfer should be preferred.

In the current study, the rate of pregnancy-induced hypertension, preeclampsia and HELLP syndrome, was 10–30%. The rate of preeclampsia was 16% in singleton, 23% in dichorionic twin, and 22% in triplet or quadruplet pregnancies. The data on triplet and quadruplet pregnancies ought to be interpreted with caution since mean time of delivery was only 28 weeks of gestation.

Preeclampsia frequently led to premature delivery. In women affected by preeclampsia, mean gestational age was 35 weeks for singleton and 33 weeks for dichorionic twins. HELLP syndrome was observed in one singleton pregnancy and one twin pregnancy, both were associated with severe preeclampsia.

Our data are consistent with a meta-analysis conducted by Storgaard et al. in 2016 that took into account 35 studies [12]. Results showed a significantly increased risk of hypertensive disorders and preeclampsia, premature birth, low birth weight, abnormal blood loss during child-birth, and a higher number of planned or emergency cesarean sections in oocyte donation pregnancies compared to IVF or ICSI pregnancies or pregnancies conceived through natural conception [12]. The data were adjusted for confounders, such as singleton versus multiple pregnancies and maternal age. The incidence of preeclampsia in singleton oocyte donation pregnancies ranged from 9.3 to 16.9% as opposed to 3.2–11.5% in IVF/ICSI and 2.4–3.8% in natural conception pregnancies. Considering twin pregnancies preeclampsia was present in 15.8–45.8% in oocyte donation pregnancies in contrast to 7.4–13.0% in IVF/ICSI pregnancies and 7.1% in naturally conceived pregnancies. Storgaard et al. calculated a two- or three-fold higher risk for the development of preeclampsia in singleton or multiple oocyte donation pregnancies than in natural conception pregnancies [12]. In developed countries hypertensive pregnancy disorders cause 16% of all maternal deaths peripartum and 25% of perinatal deaths [17]. Additionally, preeclampsia increases the risk of cardiovascular disorders later in life for both mother and child [18].

Several studies conducted on OD pregnancies support the hypothesis that abnormal placentation is induced by the immunological reaction of the mother to the genetically unknown fetus [19], a complete allograft.

Premature delivery was common with a mean gestational age at delivery of 37, 35, and 28 weeks of gestation for singleton, dichorionic twins, and triplet/quadruplet pregnancy. Interventions, including antenatal corticosteroids, tocolysis for 48 h, surgical cerclage of the cervix, pessary, or progesterone, were used in several cases. In total, premature delivery < 37 + 0 weeks of gestation accounted for 35% of singleton, 63% of dichorionic twin, and 89% of triplet/quadruplet pregnancies. 22% of triplet or quadruplet pregnancies resulted in perinatal death due to severe prematurity.

The majority of children were delivered by cesarean section. The high rate of C-sections has been described before [12]. In our clinical experience, women who have undergone oocyte donation to achieve pregnancy have a maximum need for security and therefore frequently opt for a planned C-section. However, our data show that the number of emergency C-sections exceeds the number of planned C-sections by far.

As shown above, the rate of peripartum hemorrhage with a blood loss > 500 ml was high. Severe hemorrhage with more than 1.5 l occurred in 9–29% of deliveries necessitating intensified measures. Severe blood loss is closely linked with placental pathology and uterine atony. Therefore, the high rate of placental retention is not surprising. Serena et al. detected a percentage of peripartum hemorrhage similar to ours, of 22% [20].

The above published data show a high percentage of adverse outcomes, such as IUFD accounting for 3.2% of singleton, 9% of twin, and 11.1% of triplet or quadruplet pregnancies. Perinatal death due to severe prematurity was as high as 22% of triplet or quadruplet pregnancies.

Within the group of singletons and dichorionic twins birth weight below the 10 percentile was seen in 11% and 18%, respectively. Several meta-analysis published on perinatal outcome in OD pregnancies show a significantly higher risk of low or very low birth weight, intrauterine growth retardation, preterm birth, and longer hospitalization of the neonate [21, 22].

A possible factor influencing birth weight might be the fertility procedure used. For most pregnancies, data on the exact fertility method used were not available. Studies have shown that frozen embryos result in a statistically higher birth weight than fresh embryos [23].

In light of the prohibition of oocyte donation in Germany a proportion of the German population chooses to go abroad to have fertility procedures involving donated oocytes performed. How and under which regulations these procedures are performed is out of our reach, but the German health care system still has to shoulder the potential burden associated with it.

Health care personnel in Germany should be aware of the rising number of OD pregnancies and ask their patients about the nature of their pregnancy after assuring them of their professional confidentiality. Taking into account the significantly increased risks of pregnancy-induced hypertension, preeclampsia, preterm delivery, severe peripartum hemorrhage, and placental pathology, we recommend in-depth care and monitoring of these high-risk pregnancies. Patients should be counseled about these risks and learn to recognize early signs of preeclampsia. Treating doctors and midwives should be alert for signs and symptoms of preeclampsia. According to FMF guidelines mothers at risk for preeclampsia should receive cardiovascular screening and prophylactic medication with acetylsalicylic acid [24]. Delivery should take place at a tertiary care hospital with an interdisciplinary team of health care professionals available at all times as well as a neonatal intensive care unit. Close attention should be paid to the blood loss during delivery. Obstetric care personnel should be ready to intervene as early as possible with all necessary measures to impede unnecessary additional hemorrhage.

### Strengths and limitations of this study

This paper represents the first analysis of oocyte donation pregnancies in Germany. It shows considerable risks associated with oocyte donation pregnancies and emphasizes the importance to train health care personnel properly. Due to the exceptional circumstances regarding the legal situation of oocyte donation pregnancies in Germany, we hypothesize that the above-mentioned number of OD pregnancies is only the tip of the iceberg. We assume that a number of women do not disclose information about the origin of their pregnancy due to fear of legal persecution or prejudices. Another factor might be that women who have received donated oocytes are not aware of the significantly increased pregnancy risks and therefore do not see the necessity of disclosing information on the origin of their pregnancy. On the other hand, since knowledge about oocyte donation is limited, medical personnel might not specifically ask about the mode of conception. Finally, diagnostic codes for “oocyte donation” do not exist in Germany, which impedes data analysis significantly. Therefore, the prevalence of OD pregnancies in Germany cannot be extracted from our data due to the presumably high number of unrecorded cases.

In view of the rapidly changing demographic situation in Germany these high-risk pregnancies will play an even more important role in the future.
